# Impaired neutrophil-mediated cell death drives Ewing’s Sarcoma in the background of Down syndrome

**DOI:** 10.3389/fonc.2024.1429833

**Published:** 2024-10-03

**Authors:** Serena Peirone, Elisa Tirtei, Anna Campello, Caterina Parlato, Simonetta Guarrera, Katia Mareschi, Elena Marini, Sebastian Dorin Asaftei, Luca Bertero, Mauro Papotti, Francesca Priante, Sarah Perrone, Matteo Cereda, Franca Fagioli

**Affiliations:** ^1^ Department of Biosciences, Università degli Studi di Milano, Milan, Italy; ^2^ Italian Institute for Genomic Medicine, c/o IRCCS, Candiolo, Italy; ^3^ Paediatric Oncology Department, Regina Margherita Children’s Hospital, Turin, Italy; ^4^ Department of Public Health and Paediatrics, University of Turin, Turin, Italy; ^5^ Candiolo Cancer Institute, FPO-IRCCS, Candiolo, Italy; ^6^ Pathology Unit, Department of Medical Sciences, University of Turin, Turin, Italy; ^7^ Pathology Unit, Department of Oncology, University of Turin, Turin, Italy; ^8^ Department of Oncology, University of Torino, Candiolo, Italy

**Keywords:** Ewing Sarcoma, Down syndrome, paediatric bone sarcoma, genomics, transcriptomics, neutrophils, inflammation

## Abstract

**Introduction:**

Ewing Sarcoma (EWS) has been reported in seven children with Down syndrome (DS). To date, a detailed assessment of this solid tumour in DS patients is yet to be made.

**Methods:**

Here, we characterise a chemo-resistant mediastinal EWS in a 2-year-old DS child, the youngest ever reported case, by exploiting sequencing approaches.

**Results:**

The tumour showed a neuroectodermal development driven by the EWSR1-FLI1 fusion. The inherited myeloperoxidase deficiency of the patient caused failure of neutrophil-mediated cell death and promoted genomic instability.

**Discussion:**

In this context, the tumour underwent genome-wide near haploidisation resulting in a massive overexpression of pro-inflammatory cytokines. Recruitment of defective neutrophils fostered rapid evolution of this EWS.

## Background

Down syndrome (DS) is a chromosomal abnormality characterised by Trisomy 21 (1, 2). It occurs in approximately 1 of 800 births worldwide (1) without relevant differences among races and sex (3). DS patients have an elevated risk of developing haematological disorders (4). Conversely, solid tumours are largely underrepresented in these children compared to the euploid population (5–8). Amongst the solid tumours, bone and soft-tissue sarcomas are some of the few histotypes that have been reported in these patients (8). An extremely small fraction of sarcomas consists of primary mediastinal lesions, a clinically-aggressive neoplasm with poor patient prognosis (9). These heterogeneous groups of tumours include small round blue cell sarcomas such as Ewing Sarcoma (EWS), which mainly affects children and young adults (10). This form of cancer is characterised by a recurrent chromosomal translocation that fuses an RNA-binding protein of the FET family with a transcription factor of the ETS family, with EWSR1-FLI1 being the most common somatic fusion (11).

So far, seven cases of EWS in young patients with DS (7-19 years old) have been reported and defined by cytogenetic analyses (12–16). Three tumours (45%) were driven by 11;22 translocation and underwent massive chromosomal changes (13, 14). In particular, these EWSs accumulated amplifications rather than deletions, with recurrent gains of chromosome 8 and 14 (13, 14). The authors of these studies hypothesised an involvement of the constitutional trisomy 21 in driving the disease, implicating the proto-oncogenes ETS1 and ETS2 as oncogenic drivers (13, 14). However, as they are based on cytogenetic assays, these studies lack a comprehensive molecular characterisation of EWS in DS patients.

Here, we comprehensively characterise a mediastinal EWS in a 2-year-old child with DS. Using whole exome and transcriptome sequencing, we highlight the complex genomic architecture of the EWS characterised by the clonal EWSR1-FLI1 fusion. We identify an inherited rare mutation, causative of myeloperoxidase deficiency leading to impairment of neutrophil-mediated cell death and promoting genomic instability. In this context, the tumour genome underwent near-haploidisation, resulting in a pro-inflammatory environment. Recruitment of defective neutrophils fostered fast evolution of the tumour. Our results elucidate the genetics and predisposing mechanisms of a solid tumour in a young DS patient, with possible impacts on their clinical management.

## Methods

### Sample description

The tumour used in this study was collected from the patient before chemotherapy at the Regina Margherita Children’s Hospital (Turin). The patient was enrolled in the clinical trial entitled Genomic Profile Analysis in Children, Adolescents and Young Adults with Sarcomas - SAR_GEN-ITA (ClinicalTrials.gov ID: NCT04621201). The trial was approved on 30th November 2018 by the independent ethics committee of A.O.U. Città della Salute e della Scienza di Torino - A.O. Ordine Mauriziano - A.S.L. Città di Torino (Turin, Italy), and it was conducted according to the principles of the Declaration of Helsinki and Good Clinical Practice. Parents were provided with written informed consent for the analysis and data publication.

### Fluorescence *in situ* hybridisation

Validation of EWSR1 gene translocation (22q12.2) was performed through fluorescence *in situ* hybridisation (FISH), using the ZytoLight SPEC EWSR1 Dual Color Break Apart Probe (ZytoVision GmbH, Bremerhaven, Germany) according to the manufacturer’s instructions. Red (ZyOrange, excitation 547 nm/emission 572 nm) and green (ZyGreen, excitation 503 nm/emission 528 nm) light probes targeted a proximal (chr 22:29,191,431-29,673,440) and a distal genomic (chr22:29,779,841-30,179,900) region near to the EWSR1 breakpoint. A 4 µm FFPE tumour slide was deparaffinised in xylene, de-masked using SCC (1x, pH 6) at 80°C for 20 min and digested with pepsin (0.5 mg ml−1 in 0.2 N HCl, pH 1.0; Protease and Protease Buffer II) (Abbott Laboratories, North Chicago, IL, US) for 17 min at 37 °C. Denaturation was then performed applying ten microlitres of probe onto each slide and placing them in a HYBrite (Abbott Laboratories) for 1 min at 85 °C, before overnight hybridisation at 37°C. After multiple washings and counterstaining with DAPI, FISH signals were scored with an Olympus BX61 upright microscope, using a 100x objective.

### Immunohistochemical assessment of tumour

A 3 µm slide was cut from a representative FFPE tumour block, and immunohistochemistry was performed on a Ventana BenchMark ULTRA AutoStainer (Ventana Medical Systems, Tucson, AZ, USA) with the CD99 primary antibody (O13, mouse monoclonal antibody, prediluted, incubation time: 32 minutes, Ventana, Tucson, AZ, US). Antigen retrieval was performed using the CC1 antigen retrieval buffer (pH 8.5, EDTA, 100°C, 52 min; Ventana Medical Systems, AZ, USA) and Ultraview was used to detect positivity through the chromogen 3, 3’ Diaminobenzidine (DAB). Nuclei were counterstained with Hematoxylin and Bluing reagent.

### DNA extraction and whole exome sequencing

Genomic DNA for the tumour was extracted from 10 μm-thick FFPE sections (3–6 sections per sample) using Maxwell^®^ RSC DNA FFPE Kit (Promega Corporation) on Maxwell^®^ RSC 48 Instrument (Promega Corporation), following the manufacturer’s protocol. Peripheral blood was used as a matching reference. DNA from blood samples was extracted with QIAamp DNA Blood Kit (QIAGEN), following the manufacturer’s protocol. Blood sample was considered as normal counterpart of the patient for all analyses here described. Whole exome was captured from genomic DNA for tumour and matched normal using the SureSelect XT Human All Exon V6 + COSMIC (Agilent), following the manufacturer’s protocol as previously described (17). Briefly, 0.2 μg of genomic DNA was subjected to hydrodynamic shearing by exposure to 3 minutes of sonication using a Covaris sonicator to obtain ∼200-bp-long fragments. Fragments were used to prepare libraries according to the SureSelect XT manual. Libraries were further amplified with 7–10 cycles of PCR and 150 ng were hybridised with the bait library. Captured DNA was amplified with 14 PCR cycles and barcode indexes were added. Libraries were sequenced using Illumina NovaSeq6000 in 150nt-long paired-end modality.

### Sequence alignment and variant calling

Germline and somatic mutations were identified by integrating our previously published pipeline (17) with the GATK Best Practice guidelines as implemented in the HaTSPiL framework (18). In particular, sequencing reads from each sample were aligned to the human genome reference (GRCh37/hg19) using Novoalign (http://www.novocraft.com/) with default parameters. At most, three mismatches per read were allowed, and PCR duplicates were removed using the Picard Markduplicates tool (19). To improve accuracy of variant calling, local realignment around indels was performed using GATK RealignerTargetCreator and IndelRealigner tools. Single base substitutions (SBSs) and small insertion/deletions (IDs) were identified using MuTect v.1.1.17 (20), Strelka v.1.0.15 (21) and Varscan2 v.2.3.6 (22), in tumour and normal (patient’s blood) samples independently. Only variants identified as ‘KEEP’ and ‘PASS’ in MuTect and Strelka, respectively, were considered. SBSs and InDels were retained if they (i) had allele frequency ≥5% and (ii) were in a genomic position covered by at least 10 reads.

### Identification of inherited genomic aberration

Frequency distributions of the germline heterozygous single SNVs identified by varscan2 were inspected to assess chromosome aberrations in the inherited genome of the patient. As previously proposed (17), in a diploid genome heterozygous SNVs follow a normal distribution centred around an allele frequency of 50% because both alleles occur at the same frequency in cells. In the case of allelic imbalance due to CNVs, the frequency distribution of heterozygous SNPs deviates from normality because of the unbalanced ratio between allele copies. Hence, the distribution of heterozygous SNP frequencies was used to confirm the presence of genomic alterations in the patient. To identify relevant germline mutations, we selected SNPs that harbour an allele frequency ≥25%. Clinical interpretation of germline mutations was derived from ClinVar database (https://www.ncbi.nlm.nih.gov/clinvar/) and InterVar (23), which exploits ACMG2015 guidelines (24), as previously described (25). Mutations with Combined Annotation Dependent Depletion (CADD) (26) PHREAD score higher or equal to 10 were considered ‘deleterious’. Ensembl Variant Effect Predictor (27) MaxEntScan (28, 29) was used to predict pathogenic variant effects.

### Copy number detection and purity and ploidy estimation

Somatic CNV regions were identified using Sequenza v.3.0.0 (30) with parameters window=5mb and min.reads.baf=4, keeping only positions that are covered at least by 10 reads and EXCAVATOR2 (31) with binsize=20.000 and mode=paired. To identify amplified and deleted genes, the genomic coordinates of the aberrant regions were intersected with those of 20.297 human protein coding genes of the GENCODE GRCh37 version 28 (32). A gene was considered as modified if ≥80% of its length was contained in an aberrant region. Sequenza was also used to estimate purity and ploidy values.

### Identification of cancer driver mutations

In the tumour sample, SBSs and InDels from the three different tools were identified as somatic if absent in the normal counterpart. ANNOVAR (33) was used to identify non-silent (i.e. non-synonymous, stopgain, stoploss, frameshift, non-frameshift and splicing modifications) mutations using RefSeq v.64 (http://www.ncbi.nlm.nih.gov/RefSeq/) as a reference protein dataset. SBSs and InDels falling within 2 bp from the splice sites of a gene in one of the three datasets were considered as splicing mutations. Next, a list of cancer genes was retrieved from the Network of Cancer Genes v.5 (34) (http://ncg.kcl.ac.uk/). This list was used to select 183 and 518 paediatric and adult cancer driver genes, respectively. Of these, 23 and 63 were paediatric and adult sarcoma driver genes, respectively (**Supplementary Table 8**). Furthermore, a list of 164 genes with actionable alterations was collected from the ‘PrecisionTrialDrawer’ R package (35) and these were considered as actionable genes (**Supplementary Table 8**). Genes harbouring non-silent mutations were annotated using these two gene lists. All non-silent mutations but frameshift substitutions were retained if (i) identified by at least two variant callers or (ii) occurring in genes annotated as cancer driver and/or actionable. Mutations with Combined Annotation Dependent Depletion (CADD) (26) PHREAD score higher or equal to 20 were considered as ‘highly deleterious’. CancerVar (36) was used to classify the pathogenicity of somatic variants according to AMP/ASCO/CAP/CGC 2017-2019 guidelines (37). Finally, variant frequencies were corrected by the tumour content reported by Sequenza.

### Mutational and CNV signature analysis

Mutational signature analyses were performed on all somatic mutations using SigProfilerMatrixGenerator (38) and SigProfilerExtractor (39) as previously described (40). Copy number signature analysis was performed on Sequenza results using R package ‘sigminer’ (41, 42) as previously described (41). Copy number burden was evaluated using the read_copynumber function from ​​’sigminer’ (41, 42).

### Total RNA extraction and sequencing

Total RNA was extracted from tumour biopsy using the RSC RNA FFPE Kit on Maxwell instrument. To exclude genomic contamination, total RNA was treated with DNAse I and cleared with RNA Clean and Concentration (Zymo Research). RNA quantity and quality were determined by Qubit Fluorometric Quantitation (Thermo Fisher Scientific) and using the RNA 6000 Nano kit on a Bioanalyzer (Agilent Technologies), respectively. RNA-seq library was generated from 0.1 µg of RNA using Illumina Total RNA Prep Stranded Ligation with Ribo-Zero according to manufacturer’s recommendations, and sequenced on Illumina NovaSeq6000 in 100nt-long paired-end read modality.

### Gene fusion and expression analyses of RNA-seq data

Raw sequencing reads were trimmed to avoid nucleotide overlaps between read pairs on both ends using the bbduck tool from bbmap (43) v.38.18 with parameters forcetrimright=50 and minlength=30. Trimmed reads were aligned to the human genome reference GENCODE GRCh38 version 33 (32) using STAR v.2.7.3a (44) in basic two-pass mode removing duplicates and preventing multimappings (i.e. --bamRemoveDuplicatesType UniqueIdentical and --outFilterMultimapNmax 1). Moreover, the following parameters were used: --alignInsertionFlush Right --outSAMstrandField intronMotif --outSAMattributes NH HI NM MD AS XS --peOverlapNbasesMin 20 --peOverlapMMp 0.25 --chimSegmentMin 12 --chimJunctionOverhangMin 8 --chimOutJunctionFormat 1 --chimMultimapScoreRange 3 --chimScoreJunctionNonGTAG -4 --chimMultimapNmax 20 and --chimNonchimScoreDropMin 10. Gene fusions were identified using STAR-Fusion v. 1.9.0 with options --min_FFPM 0 --FusionInspector --examine_coding_effect. Only fusions (FFPM≥0.1, LargeAnchorSupport=“YES”, LeftBreakEntropy≥1 and RightBreakEntropy≥1) were retained for further analysis. Read counts at gene level were estimated using featureCounts from Subread v. 2.0.0 (45) with parameters -O --primary -Q 1 -J -s 2 -p -B. The number of transcripts per million reads (TPM) was measured starting from the expression values of 19,923 protein coding genes.

### Ontogeny signatures evaluation

Nine signatures related to ontogeny phases (namely endoderm, mesoderm, ectoderm, ectoderm early 1, ectoderm early 2, neural ectoderm anterior, neural ectoderm posterior, neuromesodermal progenitor early and neuromesodermal progenitor late) were retrieved from two publications (46, 47). The mouse-derived ones (ectoderm early 1, ectoderm early 2, neural ectoderm anterior, neural ectoderm posterior, neuromesodermal progenitor early and neuromesodermal progenitor late) were converted to human gene symbols using the function gorth from the R package gprofiler2 v. 0.2.0 using as parameters source_organism=“mmusculus” and target_organism=“hsapiens”. Signatures were then grouped into 5 macrocategories according to their origin, namely Ectoderm (ectoderm, ectoderm early 1 and ectoderm early 2), Endoderm (endoderm), Mesoderm (mesoderm), Neuroectoderm (neural ectoderm anterior and neural ectoderm posterior) and Neuromesoderm (neuromesodermal progenitor early and neuromesodermal progenitor late). The expression in TPM of genes belonging to these categories was evaluated.

### Differential expression analysis

Gene expression data for EWS samples collected at diagnoses from three young (<4 years old) paediatric patients (i.e. SJEWS030998, SJEWS031029, SJEWS031208) available from the St.Jude Cloud (48) were retrieved under acquired accession. Raw counts were normalised as transcript per million reads (TPM), using the human genome reference GENCODE GRCh38 version 33 (32) as reference. Differential expression analysis was performed using the ‘edgeR’ R package (49), comparing the mediastinal EWS and the EWSs from the St.Jude database. P-values were corrected for multiple testing using Benjamini-Hochberg method (50). Genes that presented an absolute log2(fold change)>1 and an adjusted pvalue ≤ 0.1 were considered as differentially expressed.

### Over-representation analysis

Over-representation analyses were performed with the enricher function in the R package ‘clusterProfileR’ (51, 52) using either the 50 Hallmark, the 158 KEGG or the 278 positional gene sets defined in the mSigDb (53) and available through the R package ‘msigdbr’. Terms with p-value ≤ 0.05 were considered as significantly enriched. KEGG superfamilies of pathways were collected from the KEGG pathway databases (https://www.genome.jp/kegg/pathway.html).

### Definition of a list of neutrophil-related genes

A list of neutrophil-related genes was manually created on the basis of the work of Hendrick and Malanchi (54) (**Supplementary Table 8**).

### Deconvolution of tumour tissue cellular heterogeneity

Normalised gene expression data (TPM) of the mediastinal EWS and the EWSs available from the St.Jude database were deconvolved using xCell into 64 cell-type-specific signature (55). In particular, xCellAnalysis function from the R package ‘xCell’ (https://github.com/dviraran/xCell) was used.

## Results

### Clinical history

**Figure 1:**
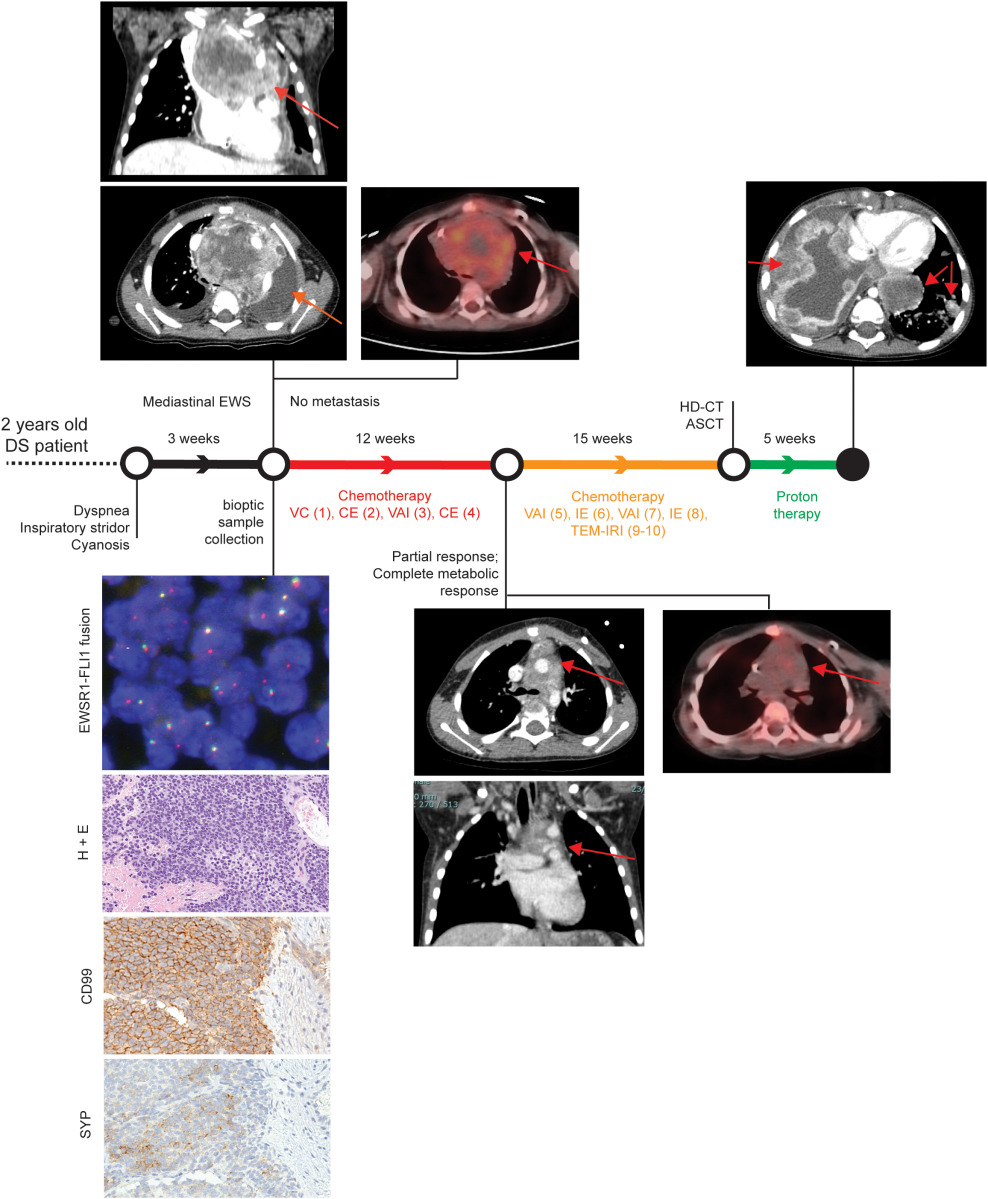
Patient clinical history. Patient history is reported with regard to diagnostic and therapeutic procedures along the time bar. Images from Thoracic CT and PET-CT at diagnosis, after first chemotherapeutic treatment, and after proton therapy (only CT) are shown. Evaluation of EWSR1 translocation t(22q12) by FISH, Hematoxylin and Eosin (H&E), and CD99 immunohistochemical images are reported in the bottom left corner. Magnification 200x. VC= Vincristine (1.4 mg/sqm) + Cyclophosphamide (850 mg/sqm). CE= Cyclophosphamide (4g/sqm) + Etoposide (600mg/sqm). VAI=Vincristine (1.4mg/sqm) + Adriamycin (90mg/sqm) + Ifosfamide (9gr/sqm). IE= Ifosfamide (9gr/sqm) + Etoposide (300mg/sqm). VAC=Vincristine (1.4mg/sqm) + Adriamycin (80mg/sqm)+ Cyclophosphamide(1.2g/sqm). TEM-IRI= Temozolomide (100mg/sqm/day) + Irinotecan (50mg/m2/day). HD-CT/ASCT = High dose chemotherapy and autologous stem cell transplantation (conditioning regimen: Treosulfan (10g/sqm/day x 3 days) + Melphalan (140mg/sqm/day x 2 days).

A two-year-old male child affected by DS presented with a three-week history of dyspnea, inspiratory stridor, and episodes of cyanosis with crying (**Figure 1**). Transthoracic echocardiographic assessment showed a retrocardiac parenchymal mass and massive pericardial effusion, with initial sign of cardiac tamponade. Urgent ultrasound-guided pericardiocentesis was required, even though complicated by a cardiac arrest. Sternotomy was then performed with evidence of tumour capsule rupture and bioptic samples of the tumour mass were collected for pathological examination. After stabilisation, total body computed tomography (CT) scan revealed a solid heterogeneous mass (8.5 cm x 8 cm x 6 cm) causing deviation of the trachea and the mediastinal vascular structures, with associated right jugular vein thrombosis (**Figure 1**, upper left panels).

Histopathological examination detected a small blue round cell tumour (**Figure 1**, left bottom panels), which was strongly positive for CD99 by immunohistochemistry, thus suggesting a Ewing Sarcoma (EWS) (11). The diagnosis of EWS was supported by identification of an EWSR1 translocation (22q12.2) using fluorescence *in situ* hybridisation (56). After informed consent signed by the parents, the patient was enrolled into the Italian paediatric sarcoma genomic study SAR-GEN_ITA, aimed at profiling inherited and somatic alterations (ClinicalTrials.gov id: NCT04621201).

A general disease staging was carried out within 72 hours from the histological diagnosis with bilateral bone marrow aspiration and positron emission tomography CT (PET-CT) scan, following the European Bone Sarcoma Guidelines (57). The results confirmed the presence of a locally advanced tumour without distant metastasis (**Figure 1**, upper left panels). A multi-agent induction chemotherapy regimen was delivered to the patient. A first chemotherapeutic cycle of Vincristine and Cyclophosphamide was tailored according to the unstable clinical condition of the patient. Due to cardiac surgical intervention, Adriamycin was omitted to avoid adjunctive toxicity. Conversely, Cyclophosphamide was considered more tolerable than Ifosfamide. After the first chemotherapeutic cycle, the patient obtained a clinical benefit with a full stabilisation of clinical condition without any new dyspnea episode. As a result, the induction treatment proceeded with three more chemotherapeutic cycles every 21 days: two cycles with Vincristine, Adriamycin and Ifosfamide and one cycle with Carboplatin and Etoposide (**Figure 1**). A complete radiological tumour response was assessed at the end of the induction period and it evidenced a partial response according to RECIST 1.1 (58) with a tumour shrinkage of 47% and a complete metabolic response at PET-CT as previously described (59, 60) (**Figure 1**, middle panels). Nevertheless, a complete surgical tumour resection was not feasible. Hence, the patient received additional chemotherapy treatment, alternating six poli-chemotherapeutic cycles every 21 days (**Figure 1**). Next, a consolidation therapy was performed, employing a high dose chemotherapy regimen with Treosulfan and Melphalan followed by autologous peripheral stem cell infusion (**Figure 1**). Again, as the complete surgical excision was non-viable, the patient received proton therapy (cumulative dose of 54 Gy in 30 fractions) as local treatment.

Despite persistent evidence of a stable and not metabolically active disease, 16 months after the initial diagnosis, the patient developed disease progression with a massive and rapidly evolving pulmonary involvement that led to patient *exitus* (**Figure 1**).

### The patient carries a rare damaging germline SNPs in the myeloperoxidase *MPO* gene

To determine inherited pathogenic predisposition of the DS patient, we performed whole exome sequencing (WES) on DNA extracted from peripheral blood reaching an average depth of coverage of 66x. We identified germline single nucleotide polymorphisms (SNPs) from sequenced reads and used such information to assess chromosomal anomalies (see Methods). To assess possible inherited changes in chromosome copies, we inspected the variant allele frequency (VAF) distribution of germline SNPs. In particular, shifts of the VAF distribution from the expected peaks of heterozygosity (VAF = 50%) and homozygosity (VAF =100%) reveal the presence of copy number changes (17). As a result, we confirmed the trisomy 21 in this patient (**Supplementary Figure 1A**).

We next sought to determine additional hereditary conditions that could be associated with, or predispose to, the onset of EWS. To do so, we focused on germline SNPs that are rare in the general population (*i.e.* minor allele frequency <0.001, see Methods), thus most likely to be associated with diseases (17). Out of 6,596 rare germline SNPs, we selected 879 defined as most likely deleterious by the Combined Annotation Dependent Depletion (CADD) algorithm (26) (i.e., CADD13 PHREAD score ≥ 10, see Methods). Of these, 17 deleterious SNPs were classified as pathogenic or as variants of uncertain significance (VUS) by at least one of two tools for clinical interpretation of genetic variants, namely ClinVar (61) and Intervar (23) (**Supplementary Table 1**). Amongst these rare deleterious SNPs, the MPO c.2031-2A>C splicing mutation was the only one reported as ‘pathogenic’ by both tools. This rare splicing mutation is known to be causative of myeloperoxidase deficiency (62). Indeed, by performing conventional splice strength analysis, we predicted a high potential to disrupt the native 3’ splice sites at intron 11 and exon 12 junction (29) (**Supplementary Figure 1B**).

### The EWS presented high mutational load and near-haploidisation

To assess the somatic alterations that characterise this mediastinal EWS, we extracted genomic DNA from tumour tissue collected at diagnosis and performed WES. We sequenced the exome at an average depth of coverage of 62x and called single base substitutions (SBSs) and small insertions/deletions (ID). We compared variant calling results between tumour and normal samples to identify somatic mutations.

**Figure 2:**
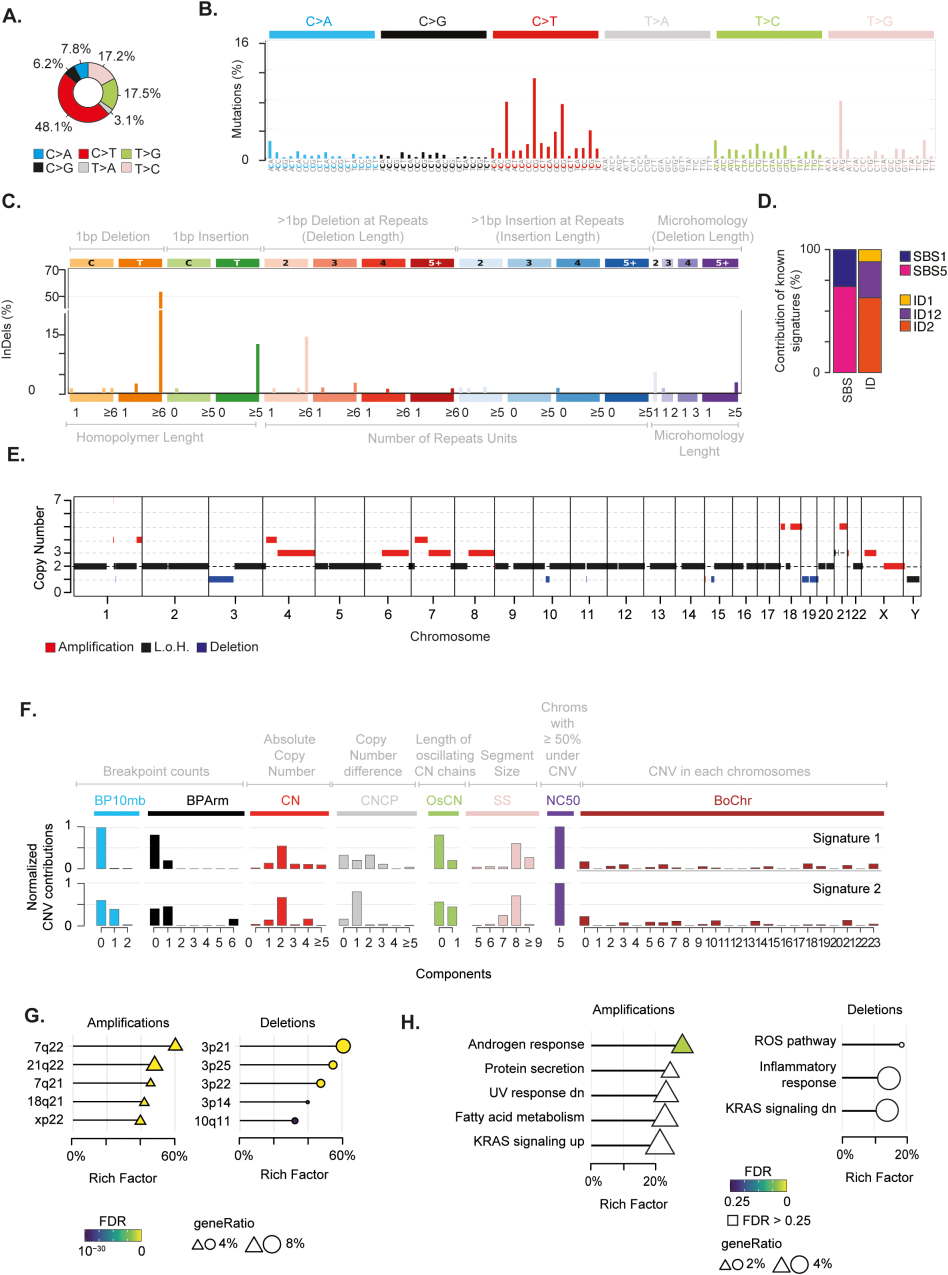
Genomic alterations characterising the mediastinal EWS. **(A)** Pie chart depicts the fraction of somatic single base substitutions (SBSs). **(B)** Most representative mutational SBS signature. **(C)** Most representative mutational ID signature. **(D)** Barplot shows the contribution of COSMIC SBS and ID signatures to the most representative signatures detected in the EWS. **(E)** Chromosomal regions undergoing somatic copy number alterations. **(F)** Most representative mutational CNV signature. BP10MB = breakpoint count per 10 Mb. BPArm = breakpoint count per chromosome arm. CN=copy number of the segments. CNCP = difference in copy number between adjacent segments. OsCN = lengths of oscillating copy number segment chains. SS = log10 based copy number segment size. NC50 = minimal number of chromosomes with 50% copy number variation. BoChr = burden of chromosome. **(G, H)** Over representation analysis performed on genes undergoing CNVs relative to chromosomal bands **(G)** and Hallmark gene sets **(H)**. Shape size indicates the fraction of CNV genes in each pathway (i.e. geneRatio). The Rich Factor represents the fraction of genes in each pathway undergoing CNVs. Colour key represents the statistical significance (FDR) of the enrichment. Only top-5 enriched pathways (FDR<0.1), if any, are shown and sorted by statistical significance.

Overall, the SBS landscape of the tumour was characterised by a prevalence of C>T and T>[C/G] substitutions (**Figures 2A, B**). C>T and T>G substitutions were in the context of G base at the immediate 3’ (*i.e.* N[C>T]G) and of A[T>G]G trinucleotides, respectively. Conversely, T>C substitutions did not present any evident design. The C>T and T>C/G mutational patterns were recapitulated by the known COSMIC SBS1 and SBS5 signatures, respectively (**Figures 2B-D**, **Supplementary Figure 1C**). Both mutational signatures have been recurrently found in paediatric cancers (40). While SBS5 aetiology is unknown, SBS1 is indicative of deamination of 5-methylcytosine (5mC) to thymine (40). The ID signatures presented a more skewed distribution, mainly characterised by single base T insertions and deletions in long thymine homopolymers, as well as small deletions in repeated regions (**Figure 2C**). This pattern recapitulated a combination of COSMIC ID2, ID12, and ID1 signatures (**Figures 2C, D**; **Supplementary Figure 1D**). ID1 and ID2 defined the single base T insertions or deletions at T stretch repeats, whereas ID12 summarised the small deletions at repeated regions (**Supplementary Figure 1D**). Similarly to SBS1, ID1 and 2 have been recurrently found in paediatric cancers and associated with DNA damage induced by replication slippage (40). Although ID12 has been previously identified in paediatric patients with brain tumours (40), its aetiology is unknown.

We then inspected the mutational landscape to identify possible driver alterations. In particular, we selected “non-silent” alterations that were likely to impair the function of the encoded protein (see Methods). These somatic variants accounted for a tumour mutational burden of 2.15, which was in the range of highly mutated paediatric tumours (63). Out of 142 non-silent mutations, we identified eight putative driver alterations (**Supplementary Table 2**). Four of them were marked as “highly deleterious” by the CADD algorithm (i.e., CADD13 PHREAD score ≥ 20) affecting known cancer driver genes. In particular, NOTCH2 H107P and BCR T1127S variants were almost clonal (i.e., present in all somatic cells), whereas EPHA7 L564F and MTOR S920F were subclonal alterations, present in around 35% of cancer cells. CancerVar classified these mutations as VUS (36). Nonetheless, NOTCH2 H107P and EPHA7 L564F predicted by CancerVar as “oncogenic” variants with the highest accuracy (i.e*.*, Oncogenic Prioritisation by Artificial Intelligence (OPAI) score > 0.84).

Next, we assessed the chromosomal status of the tumour. By profiling copy number variations (CNVs) on tumour and normal samples, we identified regions undergoing somatic alterations (see Methods). We found that 29% of the genome had undergone chromosomal changes, with the majority (22%) being amplifications (**Figure 2E**; **Supplementary Figure 1E**, **Supplementary Table 3**). Furthermore, our analysis revealed that the tumour had a ploidy of 1.4. Therefore, in absence of consistent genomic losses, these findings suggest that the tumour underwent massive genome-wide loss of heterozygosity (LOH) driven by near-haploidisation.

The analysis of copy number signatures revealed two closely related patterns characterised by (*i*) few arm and focal level breakpoints, (*ii*) a low absolute copy state with small differences between adjacent segments, and (*iii*) large alterations of approximately 100 mega base pairs hitting five chromosomes for more than 50% of their length (**Figure 2F**). To assess whether CNV localised on specific chromosome regions, we measured the over-representation of genes undergoing CNVs on 278 chromosomal bands (53). We found that 21q22 and 3p21 regions were the most enriched bands for amplified and deleted genes, respectively (**Figure 2G**, **Supplementary Table 4**). In 21q22, we detected amplification of five cancer genes, including the transcription factors ERG and RUNX1 and the RNA binding protein U2AF1. These three genes have been reported as driver genes in paediatric cancers (64). It is worth noting that amplification of 21q22 reveals the gain of one copy of the transcription factor ETS2, which has been previously suggested to play a carcinogenic role in these patients (14, 65). To identify the biological processes affected by chromosomal changes, we evaluated the over-representation of genes undergoing CNV in a list of 50 Hallmark gene sets. This list defines specific biological states displaying coherent expression (53, 66). Although not reaching stringent cutoff for multiple test correction, amplifications preferentially affected genes in the androgen response, UV response, protein secretion and metabolism of fatty acids pathways (**Figure 2H** and **Supplementary Table 5**). Conversely, deletions tended to impair genes in immune-related and reactive oxygen species (ROS) pathways. Interestingly, we found an enrichment for amplifications and deletions in genes that are known to be regulated by activation of the proto-oncogene KRAS.

Finally, to select putative drivers undergoing CNVs with the greatest accuracy, we assumed the expectation-maximisation probability that a gene belongs to a specific copy number state provided by EXCAVATOR2 (31). We identified ten genes with a probability greater than 0.9 to undergo a specific alteration (**Supplementary Table 6**). Amongst these candidate genes, we found a one-copy amplification of the proto-oncogene MET.

### The EWS derives from neuroectoderm differentiation

**Figure 3:**
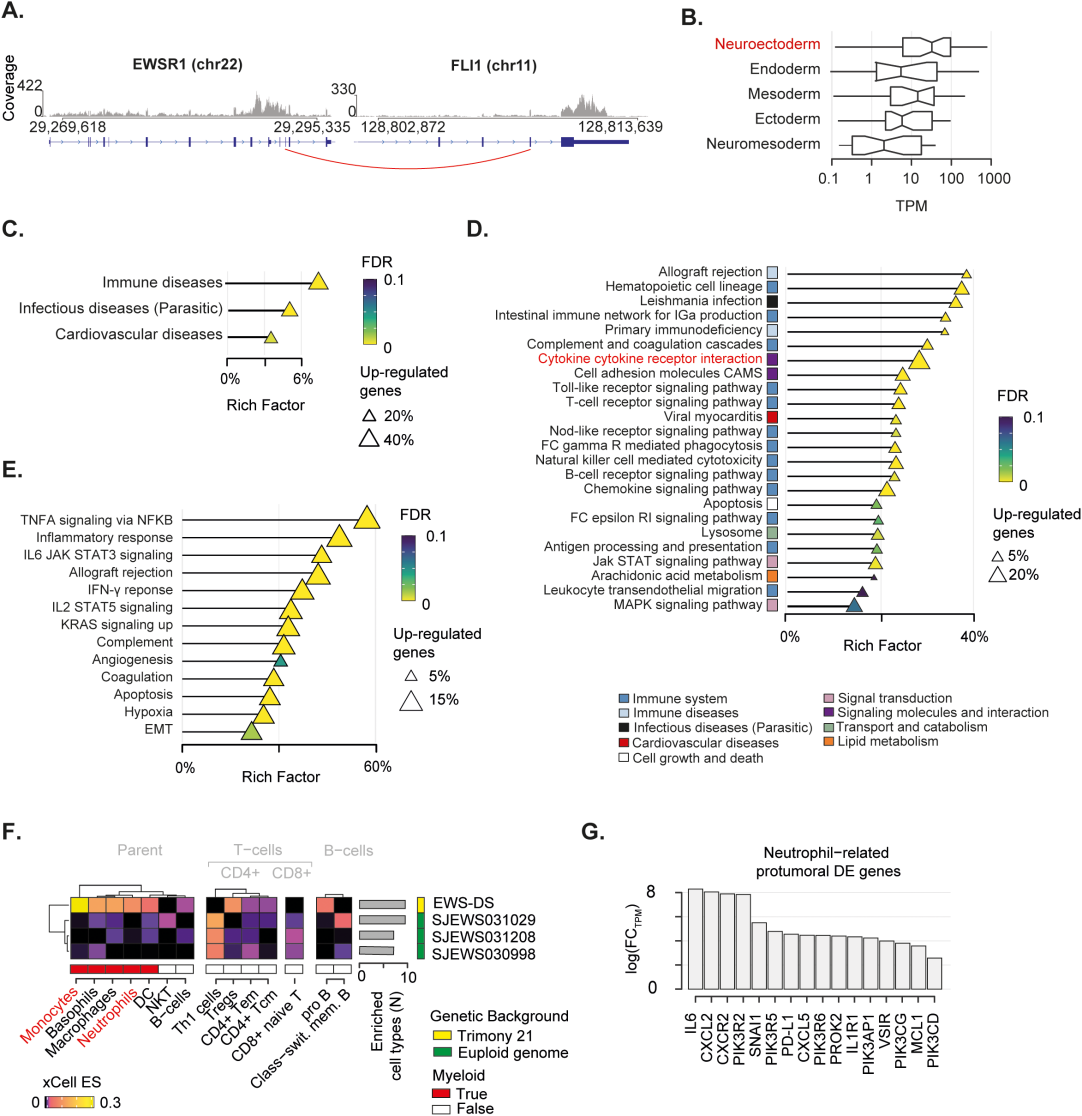
Transcriptomic landscape of the mediastinal EWS. **(A)** EWSR1-FLI1 fusion breakpoint detected by RNA-seq. Distribution of sequenced reads (i.e. coverage) is shown. Red line indicates the breakpoint of the fusion. **(B)** Boxplot depicts the cumulative normalised expression levels of genes defining embryogenesis states. **(C-E)** Over representation analysis performed on differentially expressed (DE) genes relative to KEGG superfamily of gene sets **(C)**, KEGG individual gene set **(D)**, and Hallmark gene set **(E)**. Shape size indicates the fraction of DE genes in each pathway. The Rich Factor represents the fraction of genes in a pathway that are differentially expressed. Colour key represents the statistical significance (FDR) of the enrichment. Only enriched pathways (FDR<0.1), if any, are shown and sorted by statistical significance. No enrichment found for down-regulated genes. **(F)** Heatmap shows immune-cell-specific xCell enrichment scores for the mediastinal EWS and EWSs from euploid patients. Right annotation heatmap depicts the number of enriched cell types for all tumours. **(G)** Bar chart shows fold-change in expression levels in logarithmic scale of neutrophil-related pro-oncogenic genes found as DE in the mediastinal sarcoma compared to the other EWSs.

Given the complex genomic landscape, we sought to investigate the transcriptomic profile of the EWS. To do so, we extracted total RNA from tumour tissue collected at diagnosis, and performed deep RNA sequencing (~54 Million reads). Firstly, we mapped all gene fusions and identified the EWSR1-FLI1 fusion resulting from translocation t(11,22) as the major oncogenic event (**Figure 3A**; **Supplementary Table 7**). The expression of the EWSR1-FLI1 protein induces expression of neuroectodermal differentiation markers (67). In this light, we collected five gene signatures of embryogenesis states (i.e., ectoderm, endoderm, mesoderm, neuroectoderm, neuromesoderm) (46, 47) and measured the cumulative expression of genes in these lists. The neuroectoderm signature was the most expressed compared to the others, thus corroborating the neuroectodermal origin of the EWS driven by the EWSR1-FLI1 fusion (**Figure 3B**).

### The EWS-DS microenvironment is characterised by over-represented neutrophil recruitment

To gain insight into the transcriptional programmes that characterised this mediastinal EWS, we collected gene expression data of three additional EWSs from euploid patients aged 2-3 years old, available in the St Jude database (see Methods) (48). We specifically selected children with a comparable age of the DS patient and used these data as a baseline for the gene expression comparisons. By performing differential gene expression analysis (see Methods), we identified 2,124 upregulated and 103 downregulated genes (**Supplementary Table 9**) in the EWS of the DS patient (hereafter referred as EWS-DS) compared to the other EWSs of the euploid cohort (**Supplementary Figure 2**). We then evaluated the over-representation of these differentially expressed genes in a list of 158 gene sets from the Kyoto Encyclopedia of Genes and Genomes (KEGG) (53) to identify the altered biological processes characterising the EWS-DS transcriptome. We found that up-regulated genes were significantly involved in immune- and infectious-disease-related pathways (**Figure 3C**, **Supplementary Table 10**), thus corroborating the role of inflammation in our patient. Conversely, the small number of down-regulated genes were significantly implicated in translation processes (**Supplementary Figure 2C**. **Supplementary Table 10**). By performing the over-representation analysis at single gene set level, we found that most of the significantly altered pathways had a clear connection with immune response (**Figure 3D**; **Supplementary Table 10**). In particular, a large fraction (~31%) of the cytokine–cytokine receptor interaction pathway was significantly up-regulated. This proportion accounted for more than 20% of the total differentially expressed genes, indicating a crucial impact on the activation of inflammatory response. We orthogonally evaluated the over-representation of up-regulated genes in specific biological states using the Hallmark gene sets (53, 66). Again, we found a clear enrichment of differentially expressed genes in immune related pathways (**Figure 3E**; **Supplementary Table 10**). Specifically, the majority (59%) of the tumour necrosis factor alpha (TNFA) signalling cascade activated by the NF-κB pathway was up-regulated. Similarly, a large fraction of other immune-related pro-carcinogenic pathways, such as IL6-Jak-STAT3, IL2-STAT5, and Interferon gamma (IFN-*γ*) signalling cascade, was overexpressed.

We sought to assess how this inflammatory signature reflected on the EWS-DS immune microenvironment. To do so, we deconvoluted gene expression profiles of the four tumours using xCell (55). This algorithm provides an enrichment score for each cell type in each sample, which is comparable across conditions. Out of 35 immune cell types, ten were enriched in the EWS-DS as compared to the euploid controls (**Figure 3F**). Amongst these, myeloid cells such as monocytes and neutrophils were strongly over-represented in the EWS-DS. In light of this evidence, we assess the expression levels of 34 genes that are known markers of the carcinogenic role of neutrophils (54) (**Supplementary Table 8**). Overall, 47% of these neutrophils-related carcinogenic markers were significantly differentially expressed in the EWS-DS compared to the other EWSs (**Figure 3G**). Interestingly, markers of neutrophil trafficking and recruitment during inflammation, such as *IL6*, *CXLC2*, and *CXCR2*, showed the highest fold change of expression (68, 69).

## Discussion

In this study, we extensively characterised the genetic and transcriptomic landscape of a mediastinal EWS in a two-year old patient with Down’s Syndrome. We showed that this solid tumour had developed a rare genomic architecture, probably in the background of inflammation. This condition originated from an inherited predisposition of the patient and was promoted by the tumour. Our results revealed the putative defective role of neutrophils in fostering the fast evolution of this solid tumour. Since no specific guidelines exist for the management of solid tumours in DS patients, these findings underline the need for rapid genomic screening to extend our understanding of these rare diseases and, eventually, contribute to the most appropriate clinical decisions.

Our genomic screening showed the presence of a rare pathogenic splicing variant in *MPO* (c.2031-2A>C), which is responsible for myeloperoxidase deficiency (MPOD) (62). MPOD is a primary immunodeficiency characterised by decreased MPO activity in neutrophils (62, 70). These myeloid cells are emerging as regulators of cancer development (54), especially in case of rare malignancies such as synchronous tumours (17). In physiological conditions, activated neutrophils release reactive oxygen species (ROS) and MPO to promote cell death (54). MPO regulates ROS production by catalysing the assembly of hydrogen peroxide (H_2_O_2_) with halide ions to produce hypohalous acids (71). These agents are important for MPO-mediated innate immune response. Loss of MPO leads to accumulation of H_2_O_2_, which amplifies DNA damage and activation of error-prone non-homologous end-joining repair, thereby promoting carcinogenesis (72). Therefore, impairment of neutrophil-mediated cell death driven by MPOD may have promoted carcinogenesis in the DS patient via increased genomic instability.

Our analyses on somatic alterations corroborates this scenario. We identified age-related mutational signatures (i.e., SBS1, ID1, and ID2) that characterise paediatric tumours (40). The mutational processes underlying these signatures arise from errors that are not repaired during DNA replication at mitosis (73). Specifically, the number of SBS1 substitutions mirrors how many mitoses a cell has undergone (74). Similarly, ID1 and ID2 mutational signatures result from defects in the DNA mismatch repair (40, 73). These genomic-instability-related signatures coherently describe the high mutational load of this paediatric sarcoma. Therefore, this hyper-mutability may reflect the elevated DNA damage repair levels induced by MPOD occurring during mitosis (54, 72, 75).

Driven by canonical EWSR1-FLI1 gene fusion, the EWS evidenced massive genomic instability, reaching nearly genome-wide haploidisation. This is an extremely rare phenomenon whereby the founding clone likely undergoes extensive chromosome loss during mitosis, leading to a near-haploid genome. Near-haploidisation has been reported in rhabdomyosarcoma and leiomyosarcomas, and associated with a prominent inflammatory component (76, 77). Again, the oxidative DNA damage driven by MPOD may have contributed to the catastrophic near-haploidisation of the EWS. Furthermore, somatic chromosomal losses tended to impair genes in immune-related and ROS pathways. Therefore, this finding suggests an additional impairment of inflammatory response among the surviving clones.

Genome instability is a known feature of DS patients and there is an open debate on its contribution to cancer progression (78). We found that regions of chromosome 21 and 3 (i.e., 21q22 and 3p21) were hotspots of amplified and deleted genes, respectively. The possible role for constitutional trisomy 21 in EWS development in DS patients has been hypothesised, relying on the presence of oncogenes such as ETS2 on 21q22 (14, 65). Here we found that the acquisition of one copy of the *ETS1* locus led to a significant increase of *ETS1* expression in the EWS-DS compared to other EWSs from euploid patients (FC=2.58; FDR=0.037). Furthermore, we identified the amplification of the proto-oncogene MET, a recurrent driver of resistance in multiple solid tumours (79). It has been recently shown that MET induced by tumour-derived tumour necrosis factor (TNF)-α promotes anti-carcinogenic activities in neutrophils (80). Therefore, MET amplification may have promoted the recruitment of MPO-deficient neutrophils in the microenvironment of the mediastinal sarcoma. Indeed, the tumour presented a massive overexpression of pro-inflammatory cytokines, comprising TNFA, IFN-γ, IL6-Jak-STAT3, and IL2-STAT5 signalling cascade. Furthermore, our deconvolution of immune cell infiltrates clearly shows the enrichment of neutrophils (amongst other myeloid cells) in the microenvironment of the tumour. Therefore, the crosstalk between MET amplification and TNFA, as well as IFN-γ and IL-6 pathways (68), may have fostered the recruitment of neutrophil in the tumour.

Chronic inflammation is a known feature of DS patients, driving interferonopathies and other autoinflammatory conditions (81, 82). In this patient, this baseline inflammatory condition may have been exacerbated by the predisposing splicing mutation on MPO. The inherited MPOD and acquired genomic instability may have triggered pro-inflammatory pathways in the mediastinal sarcoma. Combined with the amplification of MET, the activation of pro-inflammatory signals fostered the recruitment of MPO-impaired neutrophils, which probably could not have promoted cell death. Eventually, this condition may have had a role in the final chemoresistance and *exitus* of the patient.

## Data Availability

The data presented in the study are deposited in the Sequence Read Archive (SRA) repository, accession number PRJNA1136445.

## Supplementary material

Supplementary Figure 1
**(A)** Density distribution of mutational frequency of germline variants. Chr1-20 and chr22 are depicted in grey, while chr21 is depicted in pink. **(B)** Bar chart shows MaxEntScan score for canonical splicing site (red) and mutated one (black). **(C)** Deconvolution of SBS patient signature with respect to annotated Cosmic profiles (SBS5 and SBS1). **(D)** Deconvolution of InDels patient signature with respect to annotated Cosmic profiles (ID2, ID12 and ID1). **(E)** Genome view represents variant allele frequencies of single base mutations and the depth ratio profile. Red horizontal lines represent mean value considering genome segments optimised by Sequenza.

Supplementary Figure 2
**(A)** Principal component analysis represents transcriptional distribution of the report case (i.e., “Mediastinal EWS”) and three EWS samples from young euploid children of the St Jude cohort (indicated as “St Jude”). **(B)** Heatmap shows correlation between gene expression in the different patients **(C)** Over-representation analysis performed on downregulated genes relative to KEGG gene sets.
