# Supplementary figures and images for: Impaired neutrophil-mediated cell death drives Ewing’s Sarcoma in the background of Down syndrome
Source: Front Oncol. 2024 Oct 3;14:1429833. doi: 10.3389/fonc.2024.1429833 (PMC11484044; doi:10.3389/fonc.2024.1429833)

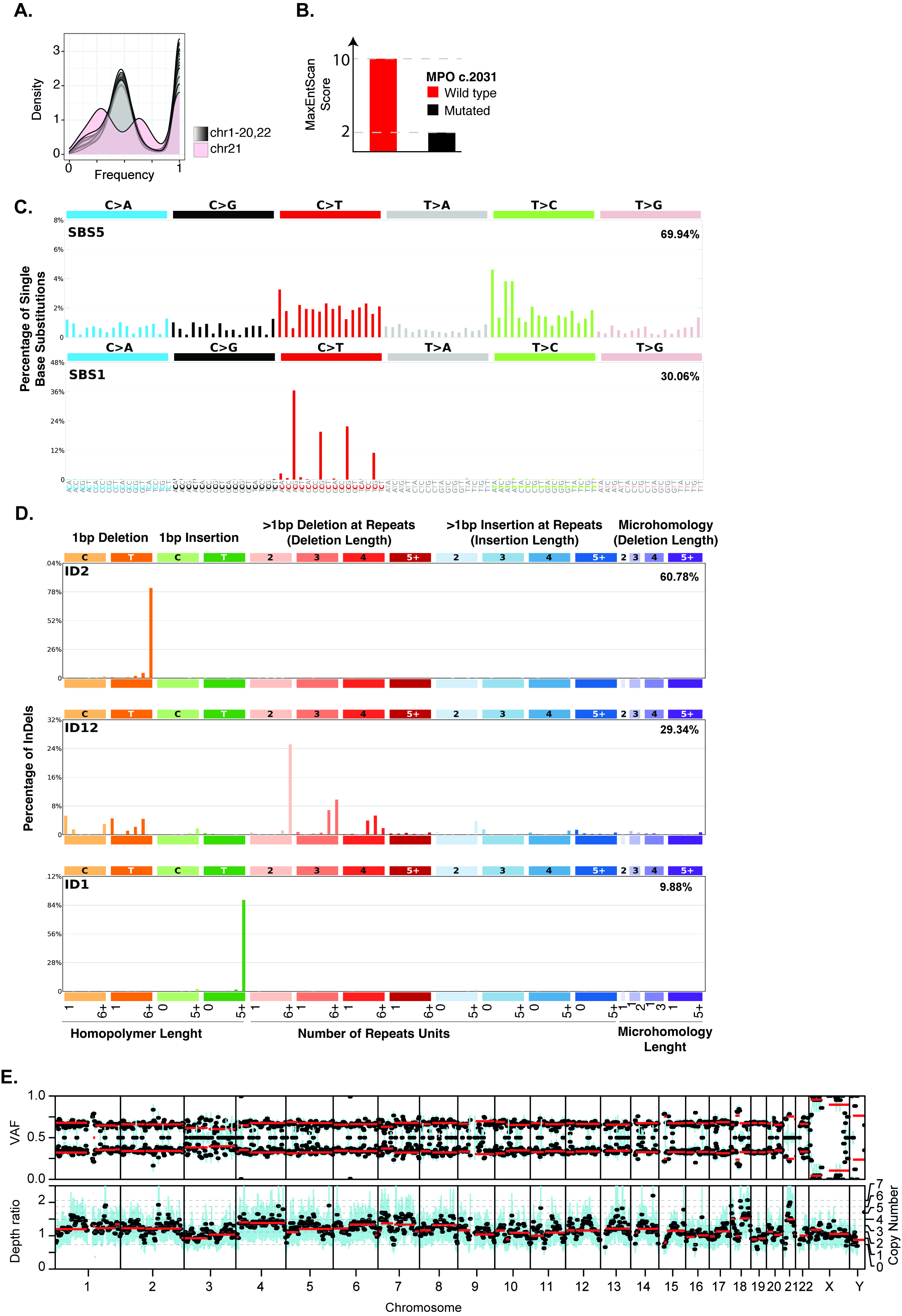

Supplement: Supplementary Figure 1 — (A) Density distribution of mutational frequency of germline variants. Chr1-20 and chr22 are depicted in grey, while chr21 is depicted in pink. (B) Bar chart shows MaxEntScan score for canonical splicing site (red) and mutated one (black). (C) Deconvolution of SBS patient signature with respect to annotated Cosmic profiles (SBS5 and SBS1). (D) Deconvolution of InDels patient signature with respect to annotated Cosmic profiles (ID2, ID12 and ID1). (E) Genome view represents variant allele frequencies of single base mutations and the depth ratio profile. Red horizontal lines represent mean value considering genome segments optimised by Sequenza. [file Image1.tif]

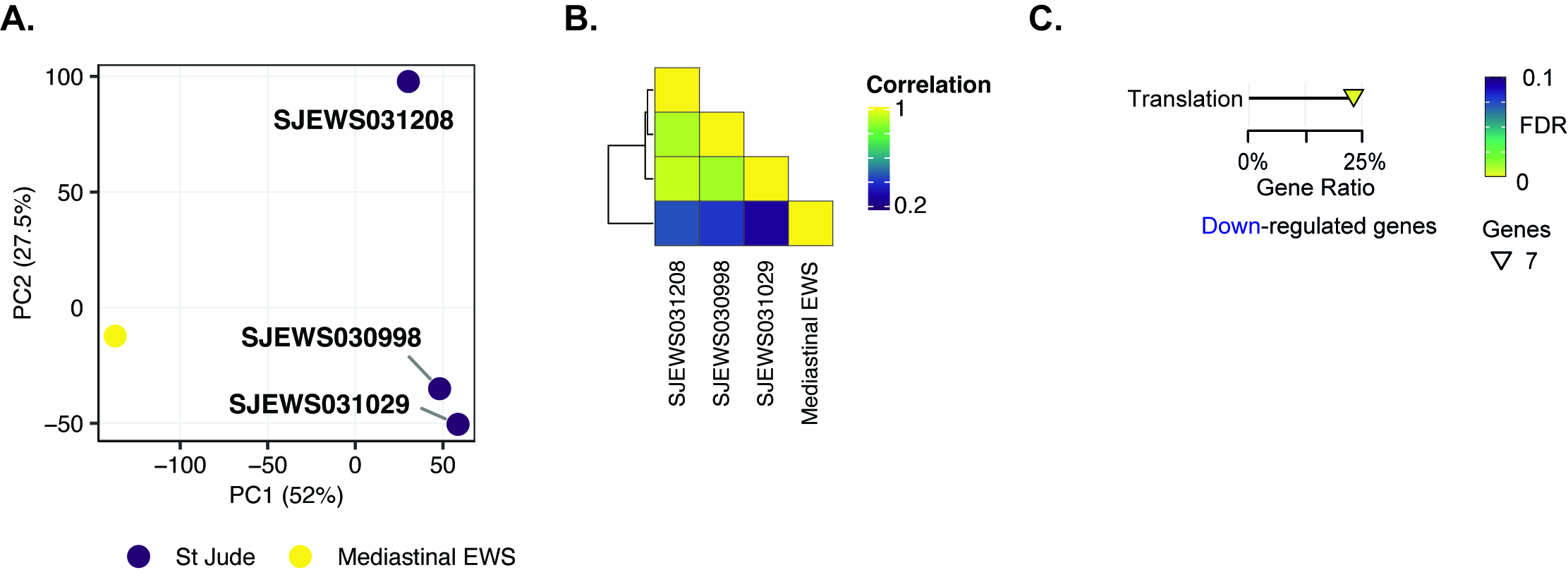

Supplement: Supplementary Figure 2 — (A) Principal component analysis represents transcriptional distribution of the report case (i.e., “Mediastinal EWS”) and three EWS samples from young euploid children of the St Jude cohort (indicated as “St Jude”). (B) Heatmap shows correlation between gene expression in the different patients (C) Over-representation analysis performed on downregulated genes relative to KEGG gene sets. [file Image2.tif]
